# What’s in an eye roll? It is time we explore the role of workplace incivility in healthcare

**DOI:** 10.1186/s13584-018-0209-0

**Published:** 2018-03-14

**Authors:** Sharone Bar-David

**Affiliations:** Bar-David Consulting, 49 Fairleigh Crescent, Toronto, ON M6C 3S1 Canada

**Keywords:** Workplace incivility, Respectful workplace, Rudeness, Eye rolling, Passive aggressive behavior, Workplace violence, Gasut ruach, Healthcare, Iatrogenic injury

## Abstract

A recent study of patient violence toward hospital physicians and nurses offers a welcome perspective in its classifying of aggressive behaviors as workplace violence. While patients and families are widely recognized as sources of rude behaviors, we need to shed light on passive aggressive and other low-level rude behaviors that take place frequently amongst hospital personnel as well. Studied under the term “workplace incivility,” these seemingly insignificant behaviors that show lack of regard for colleagues have far reaching negative consequences. Examples of such consequences include intentionally reducing work effort, spending time worrying, and taking frustration out on customers. In addition, incivility creates a spiral effect, where one type of incivility breeds other forms of same. In healthcare, rudeness plays a pivotal role due to its negative impact, which goes to the heart of service delivery. For example, healthcare professionals who are exposed to incivility, even when not directed specifically at them, are at risk of inflicting iatrogenic injury. Within the complexity of hospital environments, incivility gets fueled and maintained by underlying beliefs such as “because we work in a high-pressure environment, it’s okay to skip the niceties.” Tackling these beliefs is key to taming workplace incivility. This article poses questions worthy of further scientific inquiry. Finally, Israeli researchers and practitioners are advised to find a better term for workplace incivility to replace the currently used, excessively negative term *gasut ruach.*

In an enlightening article [1], Sigal Shafran-Tikva and her colleagues examine different types of violence that nurses and physicians in a Jerusalem hospital experience, directed at them from patients and their companions. The researchers found high levels of exposure to violence in the six months that preceded the study, ranging from 52% to 96%. Rates varied based on seniority, department, and years of experience.

Workplace violence research does not usually encompass low-intensity negative behaviors. This study offers a fresh contribution in that it classifies passive aggressive behaviors as workplace violence. Examples in the study included sharp looks, stern facial expressions, and muttering. The study included the passive aggressive category after participants in preliminary focus groups mentioned such behaviour frequently. In a companion study [2], focus group members viewed such conduct as a contributing factor to the unfolding of violent events.

Passive aggressive and other forms of low-level rude behaviors occur not only in the interface between patients and staff. They are an ever-present component in the relationships amongst hospital personnel themselves. This mistreatment of coworkers has in the past two decades been studied under the term “workplace incivility.”

Workplace incivility refers to the seemingly insignificant behaviors that are rude, disrespectful, discourteous, or insensitive, where the intent to harm is ambiguous or unclear [3]—the insensitive behaviors that display a lack of regard for others, in violation of workplace norms for mutual respect [4]. It happens between peers, or from a superior toward someone lower in the hierarchy, or directed at a superior. Typical examples include belittling comments or dismissive gestures (eye rolling, lip sounds, sighs, muttering), skipping greetings, gossip, social exclusion, silent treatment, sarcasm, and even the rude use of mobile devices.

It is no secret that workplace incivility is an ongoing challenge in the healthcare sector. In the operating room the surgeon curtly reprimands a nurse who handed the wrong instrument. In the nursing station, a nurse rolls his eyes at a colleague’s story. And in the corridor, a social worker makes a disparaging comment to the cleaner in front of a patient’s family. When daily work involves life and death, small niceties easily fall by the wayside. The task at hand tends to trump attention to relationships, and ever-present stress triggers discourteous behaviour. Furthermore, when jobs are performed in tight physical proximity and there is high role interdependence, there is little time or space for people to check themselves before behaving badly, and even less breathing space to control their reactiveness to a colleague’s rude behaviour before spiralling into a response that in itself is uncivil.

Workplace incivility has far-reaching negative effects. Research from across seventeen industries shows that 48% of respondents who were asked about their reaction to incivility reported that they purposely lowered their work effort. Eighty percent of respondents said that they lost time worrying about the incident, and 66% reported that their performance declined following an incivility incident. As many as 25% admitted they took out their frustration on a customer or client after an incivility event [5]. To this point, in another article published previously in this journal [6], Shafran-Tikva and her colleagues found that 39% of 4047 hospital staff respondents perceived that their own uncivil behavior contributed to the unfolding of violence incidents. Finally, research of health professionals has found that having an uncivil colleague or supervisor exacerbates mental and physical health problems [7].

Moreover, incivility breeds incivility. It has a spiral effect, which can spawn more serious aggressive behaviors [4]. And like the common cold, low-intensity negative behaviors spread easily from one person to another [8]. To boot, initial evidence suggests that even witnessing rudeness enacted by an authority figure decreases observers’ performance on creative tasks [9].

Acknowledging that any one of us can engage (albeit unintentionally) in workplace incivility is key to affecting change in the workplace. We can be curt on a bad day. Or express frustration in the form of an eye roll, a mutter, or sigh. My experience as a trainer and consultant demonstrates that once you get people to own up to the fact that we are all culpable in some way, and invite them to lead by (good) example, change becomes possible. But when people don’t see this in themselves, they fall into finger-pointing, self-righteousness and feeling victimized by others’ rudeness. Judging and unforgiving, they bear grudges (sometimes for years!) and get even. None of this is good for workplace culture, let alone service delivery.

Another key to taming workplace incivility is tackling the underlying beliefs that enable it in the first place. Much of incivility—especially in hospitals—is fueled by potent core notions that go unnoticed and unquestioned, leading to implicit justification for uncivil behavior. Prevalent beliefs in healthcare include: because we work in a high-pressure environment, it’s okay to skip the niceties; people shouldn’t be so sensitive—if you want to enter the kitchen you have to tolerate the heat; no one can hold physicians accountable for abrasive conduct; we’re like a family here—we don’t have to watch every word we say to each other, and; the best way to release steam when you’re frustrated with someone is to vent about them to another colleague. With these beliefs pulsating in the background, it is as if everyone has blinders on, preventing them from seeing uncivil behavior for what it is.

Israeli researchers have taken a lead role in exploring what happens when professionals are exposed to rudeness from external players rather than coworkers, in some cases not even aimed directly at them. Most exciting, much of their work focuses on the healthcare sector. In one study, twenty-four NICU teams participated in a training simulation where they were to diagnose and treat a preterm infant whose condition deteriorated rapidly. Half the teams were exposed at the outset to general negative comments from a “foreign expert” regarding healthcare in Israel. They found the experience of incivility reduced the amount of information sharing and help seeking behaviors between team members who were exposed to the foreign expert’s comments. This led to poor team diagnostic and procedural performance, to the extent of possibly leading to iatrogenic injury [10]. In another interesting exploration, Amir Erez of the University of Florida is currently examining the role of exposure to rudeness in physicians’ diagnostic anchoring, that is, tracing the extent to which being exposed to rudeness leads physicians to stick to an initial diagnosis and failing to adjust this initial diagnosis in the face of additional conflicting information.

A word of caution: Using the English term workplace incivility as the common phrase to describe those seemingly inconsequential rude behaviours has been very helpful to those of us who work with organizations in training and consulting capacities—it is easy for people to admit that their own behaviour can at times be uncivil. However, in Israel the current term for workplace incivility— *gasut ruach—*is problematic. While is refers to rudeness and disrespect, its additional nuances of roughness and crudeness make the term excessively negative and charged with undesirable connotations. Most people would be hard-pressed to admit, even to themselves, that they engage in *gasut ruach*, even if only fleetingly. This in turn makes workplace interventions more challenging. Selecting a more neutral, nuanced Hebrew term will be more useful in the long run. One contender might be *cho’sser de’rech e’retz* (lack of “*derech eretz”*). Rooted in Talmudic teachings, *derech eretz* refers to a desired mode of proper behavior of courtesy, respect, politeness, and good manners—basic human decency that shows reverence and adherence to community norms for appropriate behavior. Be it *derech eretz* or perhaps another term, I urge Israeli researchers to find a phrase that will be more useful to those of us in the trenches trying to affect change.

Shafran-Tikva and her colleagues suggest a revisit of the role that passive aggressive behavior plays in violent episodes. This is indeed a worthy area to explore. Other directions for further exploration include:

- The relationships between inter-staff incivility and patient/family incivility. How does rudeness among staff members affect the levels of respect and courtesy with which they treat patients, and vice versa?
- Effects on quality of care and patient outcomes. How does incivility affect service, and outcomes? What role do professional discipline, and cultural background, play in this regard?
- Effects on health and safety. Rudeness is upsetting, and upset people can’t think straight. How does rudeness impact health and safety risks such as mistakes in administering medication, unsafe handling of equipment, compromised infection control, and increased workplace accidents?
- Effects on decision-making. How do rudeness and incivility affect the quality of decision making in healthcare? What role do distraction and upset play?
- Effects on innovation. Innovation can only happen when people trust that their half-baked ideas will be met with open minds. This can’t happen when one fears even subtle belittling or dismissiveness. We need to learn more on this front.
- The effectiveness of incivility training interventions. Which interventions work best, when, and how?

## Conclusion

The case against incivility in healthcare is strong. Scientific inquiry has only begun scrapping the surface of the malignant tentacles that rudeness sends into the workplace, the people who inhabit it, and the patients and families who are at the core of it all. It is high time we give this topic its due attention.
